# The Cyclopædia of Practical Medicine

**Published:** 1834-04-01

**Authors:** 


*The Cyclopædia of Practical Medicine.* Edited by John Forbes, m.d., Alexander Tweedie, m.d., and John Conolly, m.d. Part XX.—London, 1834. 8vo. pp. 128.

The present number commences with an article by Dr. Carswell, on *the Softening of Organs.* He is of opinion that the perforation of the stomach, which has been elevated into the rank of a new disease by the French pathologists, is in fact caused by the gastric juice after death. He says:

"From all the facts brought forward on this part of our subject the following principles may be established:

"1st. That the softening, erosion, and perforation of the walls of the stomach, attributed by the greater number of pathologists to morbid conditions of this organ, may be produced, whatever may be their form, degree, extent, or situation, by the gastric acid.

"2d. That no pathological condition of the stomach or of any other organ is necessary to the production of these lesions.

"3d. That all of them are met with in individuals who, in the full enjoyment of health, are suddenly deprived of life; and in those who die from various diseases.

"4th. That all of them are met with, after death, in healthy and diseased stomachs, which contain gastric acid.

"5th. That they are produced by introducing this fluid into a healthy dead stomach.

"6th. That the varieties observed in the form, degree, extent, and seat of these lesions, depend on modifications of the gastric acid, the action of which on the stomach is regulated by a certain number of physical conditions in which this organ may be placed.

"7th. That softening, erosion, and perforation from the action of the gastric acid, are observed in other organs besides the stomach, viz. in the œsophagus and intestines, from the direct communication which exists between them and the former organ; and in the peritoneum, liver, spleen, diaphragm, pleuræ, and lungs, in consequence of the perforation of the stomach and œsophagus.

"8th. That all these lesions of the stomach, intestines, and of the other organs, are produced after death." (P. 20.)

We next come to a paper by Dr. Prichard, on *Somnambulism and Animal Magnetism:* it is well written; but, as we are about to notice Mr. Colquhoun's book on this subject, we shall indulge ourselves with only one extract, describing a most singular case of ecstasis.

"A gentleman, about thirty-five years of age, of active habits and good constitution, living in the neighbourhood of London, had complained for about five weeks of slight headach. He was feverish, inattentive to his occupations, and negligent of his family. He had been cupped and had taken some purgative medicine, when he was visited by Dr. Arnould, of Camberwell, who has favoured us with the following history. By that gentleman's advice he was sent to a private asylum, where he remained about two years; his delusions very gradually subsided, and he was afterwards restored to his family.

"The account which he gave of himself was almost verbatim as follows. We insert the statement as we received it from his physician. 'One afternoon in the month of May, feeling himself a little unsettled and not inclined to business, he thought he would take a walk into the city to amuse his mind; and having strolled into St. Paul's Church-yard, he stopped at the shop-window of Carrington and Bowles, and looked at the pictures, among which was one of the cathedral. He had not been long there before a short grave-looking elderly gentleman, dressed in dark brown clothes, came up, and began to examine the prints, and occasionally casting a glance at him, very soon entered into conversation with him; and, praising the view of St. Paul's which was exhibited at the window, told him many anecdotes of Sir Christopher Wren the architect, and asked him at the same time if he had ever ascended to the top of the dome. He replied in the negative. The stranger then inquired if he had dined, and proposed that they should go to an eating-house in the neighbourhood, and said that after dinner he would accompany him up St. Paul's: 'it was a glorious afternoon for a view, and he was so familiar with the place that he could point out every object worthy of attention.' The kindness of the old gentleman's manner induced him to comply with the invitation, and they went to a tavern in some dark alley, the name of which he did not know. They dined, and very soon left the table, and ascended to the ball just below the cross, which they entered alone. They had not been there many minutes, when, while he was gazing on the extensive prospect, and delighted with the splendid scene below him, the grave gentleman pulled out from an inside coat-pocket something like a compass, having round the edges some curious figures; then having muttered some unintelligible words, he placed it in the centre of the ball. He felt a great trembling and a sort of horror come over him, which was increased by his companion asking him if he should like to see any friend at a distance, and to know what he was that moment doing, for if so, the latter could show him any such person. It happened that his father had been for a long time in bad health, and for some weeks past he had not visited him. A sudden thought came into his mind, so powerful that it overcame his terror, that he should like to see his father. He had no sooner expressed the wish than the exact person of his father was immediately presented to his sight on the mirror, reclining in his arm-chair, and taking his afternoon sleep. Not having fully believed in the power of the stranger to make good his offer, he became overwhelmed with terror at the clearness and truth of the vision presented to him; and he entreated his mysterious companion that they might immediately descend, as he felt himself very ill. The request was complied with; and on parting under the portico of the northern entrance, the stranger said to him, 'Remember, you are the slave of the man of the mirror!' He returned in the evening to his home, he does not know exactly at what hour; felt himself unquiet, depressed, gloomy, apprehensive, and haunted with thoughts of the stranger. For the last three months he has been conscious of the power of the latter over him.' Dr. Arnould adds, 'I inquired in what way his power was exercised? He cast on me a look of suspicion mingled with confidence; took my arm, and, after leading me through two or three rooms, and then into the garden, exclaimed, 'It is of no use, there is no concealment from him, for all places are alike open to him, he sees us and he hears us now.' I asked him where this being was who saw and heard us? He replied, in a voice of deep agitation, 'Have I not told you that he lives in the ball below the cross on the top of St. Paul's, and that he only comes down to take a walk in the church-yard and get his dinner at the house in the dark alley. Since that fatal interview with the necromancer,' he continued, 'for such I believe him to be, he is continually dragging me before him on his mirror, and he not only sees me every moment of the day, but he reads all my thoughts, and I have a dreadful consciousness that no action of my life is free from his inspection, and no place can afford me security from his power.' On my replying that the darkness of the night would afford him protection from these machinations, he said, 'I know what you mean, but you are quite mistaken. I have only told you of the mirror, but in some part of the building which we passed in coming away, he shewed me what he called a great bell, and I heard sounds which came from it, and which went to it; sounds of laughter, and of anger, and of pain; there was a dreadful confusion of sounds, and as I listened with wonder and affright, he said, 'This is my organ of hearing; this great bell is in communication with all other bells within the circle of hieroglyphics, by which every word spoken by those under my control is made audible to me.' Seeing me look surprised at him, he said, 'I have not yet told you all; for he practises his spells by hieroglyphics on walls and houses, and wields his power, like a detestable tyrant as he is, over the minds of those whom he has enchanted, and who are the objects of his constant spite, within the circle of the hieroglyphics.' I asked him what these hieroglyphics were, and how he perceived them? He replied, 'Signs and symbols which you in your ignorance of their true meaning have taken for letters and words, and read as you have thought: Day and Martin and Warren's blacking. Oh! that is all nonsense! they are only the mysterious characters which he traces to mark the boundary of his dominion, and by which he prevents all escape from his tremendous power. How have I toiled and laboured to get beyond the limits of his influence! Once I walked for three days and three nights, till I fell down under a wall exhausted by fatigue, and dropped asleep; but on awakening I saw the dreadful signs before my eyes, and I felt myself as completely under his infernal spells at the end as at the beginning of my journey.'

"It is probable that this gentleman had actually ascended to the top of St. Paul's, and that impressions there received being afterwards renewed in his mind when in a state of vivid excitement, in a dream of ecstatic reverie, became so blended with the creations of fancy as to form one mysterious vision, in which the true and the imaginary were afterwards inseparable. Such at least is the best explanation of the phenomena that occurs to us." (P. 37.)

DR. BISSET HAWKINS, in an article on *Medical Statistics*, says, "The proportion of births to a marriage fluctuates in different countries: in England it is about four births to a marriage; at Paris the proportion is scarcely 2.44 births to a marriage; while in some villages in Scotland it is so high as seven." We doubt if the proportion varies much in different countries, though it may in different towns or villages. Throughout Europe, we believe, the proportion is four and a fraction to a marriage; it is the fraction only which varies.

DR. WILLIAMS has an account of the *Stethoscope*, considered as an instrument; the diagnostic signs derived from it having been discussed under the head *Auscultation*. The following remarks will interest many of our readers:

"Our limits do not permit us to describe other forms of stethoscopes that are to be met with. In all those which we have seen acoustic rules are sacrificed to portability or elegance. That of M. Piorry, which is commonly used, is faulty in having the conducting power of the wood impeded by screws and a thick cap of ivory; besides which, the excavated end is generally very ill fitted. Although, when unscrewed, more portable than the other instrument, the trouble of screwing and unscrewing the several parts is enough to counterbalance this advantage. Our friend Dr. Stroud uses a caoutchouc tube, with an ivory funnel attached, like the flexible hearing-trumpet. The flexibility of this instrument is certainly an advantage, but it is obtained at a great sacrifice of intensity and distinctness of the sounds, in consequence of the imperfect and irregular reflecting power of the interior of the tube. This flexible stethoscope separates the sound from the impulse of the heart more completely than any other. Although, for general purposes, the common stethoscope fulfils sufficiently well its triple office, it might be well for the attainment of greater accuracy in physical diagnosis, if auscultators in hospitals would use separate instruments for the three classes of signs. 1. A solid cylinder of wood for the auscultation of the heart. 2. A metallic tube, half an inch in diameter, furnished with a wood or ivory ferule at the pectoral, and an ear-piece at the other end, for the investigation of the respiration and vocal resonance of small spots. 3. A metallic tube, like the last, but with its pectoral end expanded into a tapering cone, for the same purposes as the stethoscope without the stopper. After a little practice, we have little doubt that these instruments would be more powerful aids than the common stethoscope, but we do not pretend to recommend them for general adoption." (P. 68.)

Dr. A. T. Thomson, in a judicious paper on *Stimulants*, recommends the use of the ioduret of iron. After observing, that the iodurets of mercury and lead must be given even in smaller doses than iodine itself, he says:

"On the other hand, the combination with iron, which was introduced to the notice of the profession by the writer of this article, diminishes the irritative action of the iodine, whilst the iron being rendered soluble, and in a state to be readily converted into the protoxide, is carried into the habit with the iodine, and aids its deobstruent influence by giving tone and support to the system.* It is admirably adapted for chlorotic affections, and cases of glandular obstructions, connected with diminished power and a leucophlegmatic state of the habit. On account of its deliquescent property it cannot be administered in substance; and when dissolved it is converted into a hydriodate, in which form it may be administered in doses of from two to six grains three times a day. The influence of both its components is rapidly visible on the habit by the improved colour of the skin, the increase of appetite, exhilarated spirits, and invigorated strength of the patient; and so quickly does it get into the system, that in twenty-four hours after the first dose has been taken, both the iron and the iodine can be detected in the secretions. It is incompatible in prescriptions with alkalies and their carbonates; the metallic salts; all vegetable infusions and decoctions containing tannin and gallic acid; the preparations of opium, henbane, and conium; the alkaloids and their salts, and chlorine. It has one advantage over all the other preparations of iodine, it does not produce emaciation, or that wasting of glandular bodies, which renders the closest watchfulness requisite in their administration." (P. 79.)

Several other articles merit commendation; and, on the whole, this number is a good one.

We must not omit to mention, that the editors say that a fourth volume "is found necessary to the completion of the undertaking." Thus it is that the illaudable practice of publishing books in fragments leads to prospectus-breaking.

* "The ioduret of iron is prepared by placing one part of soft tempered iron-wire in a hollow porcelain vessel with a considerable quantity of distilled water, and adding five parts, by weight, of pure iodine, and then subjecting the mixture to heat, constantly stirring until the solution is accomplished and the liquid is nearly clear. The solution is then to be filtered and immediately evaporated to dryness in a flask, which must be broken as soon as the ioduret has crystallized, and the preparation directly put into a well-stopped phial. It is a proto-ioduret, containing one equivalent of each of its components. When well made, and well preserved from the air, it dissolves entirely, and affords a pale greenish-yellow solution; but when not well preserved from the air, a portion of the iron is converted into the peroxide of the metal, and a sesqui-ioduret is formed, so that when it is rubbed up with water the peroxide remains insoluble."

